# Frequency of Hepatitis C in hospitalized patients with chronic kidney disease

**DOI:** 10.12669/pjms.331.11553

**Published:** 2017

**Authors:** Salman Tahir Shafi, Muhammad Zaigham Hassan, Mohammed Saleem, Roshina Anjum, Wajid Abdullah, Tahir Shafi

**Affiliations:** 1 Dr. Salman Tahir Shafi, Associate Professor of Nephrology, Department of Nephrology, Sharif Medical and Dental College, Sharif Medical City Road JatiUmra, Lahore, Pakistan; 2 Dr. Muhammad Zaigham Hassan, Post graduate Resident, Department of Nephrology, Sharif Medical and Dental College, Sharif Medical City Road JatiUmra, Lahore, Pakistan; 3 Dr. Mohammad Saleem, Post graduate Resident, Department of Nephrology, Sharif Medical and Dental College, Sharif Medical City Road JatiUmra, Lahore, Pakistan; 4 Dr. Roshina Anjum, Post graduate Resident, Department of Nephrology, Sharif Medical and Dental College, Sharif Medical City Road JatiUmra, Lahore, Pakistan; 5 Dr. Wajid Abdullah, Post graduate Resident, Department of Nephrology, Sharif Medical and Dental College, Sharif Medical City Road JatiUmra, Lahore, Pakistan; 6 Prof. Dr. Tahir Shafi, Department of Nephrology, Sharif Medical and Dental College, Sharif Medical City Road JatiUmra, Lahore, Pakistan

**Keywords:** Hepatitis C, Chronic Kidney Disease, Frequency

## Abstract

**Background and Objectives:**

Hepatitis C and chronic kidney disease (CKD) are major global health problems and are highly prevalent in Pakistan. There is limited information on prevalence of hepatitis C in patients with CKD not yet on dialysis. The objective of this study was to determine the frequency of hepatitis C in hospitalized chronic kidney disease patients at a tertiary care center in Pakistan.

**Methods:**

The study design was cross-sectional in nature. Patients between ages of 20-80 years with CKD not previously on renal replacement therapy and who were admitted to nephrology ward at a tertiary care facility were included. Hepatitis C was tested using 3^rd^ generation enzyme linked immunosorbent assay (ELISA). Hepatitis C RNA was tested by polymerase chain reaction (PCR) in patients with positive ELISA.

**Results:**

A total of 180 patients were included in the study. Mean age of patients was 48.7±14.9 years. Of all patients, 105 (58.3%) were males and 75 (41.7%) were females, 152 (84.4%) had hypertension, 113 (62.8%) had diabetes mellitus and 26 (14.9%) had known cardiovascular disease. Mean eGFR of patients was 11.4±9.4 ml/min/1.73 m2. Of all patients with CKD, 49 (27.2%) had hepatitis C test positive by ELISA. Hepatitis C PCR testing was done in 39 patients with hepatitis C ELISA positive status and 29 (74.4%) tested positive. Risk factors and clinical characteristics of patients with and without positive hepatitis C antibody by ELISA were similar.

**Conclusion:**

A significant proportion of hospitalized CKD patients have hepatitis C. Strict universal infection control measures should be implemented in nephrology wards to prevent transmission of hepatitis C infection.

## INTRODUCTION

Both Hepatitis C and chronic kidney disease (CKD) are major global public health problems. It is estimated that worldwide more than 185 million people have hepatitis C. Of these patients, 50-85% develop chronic infection.^1^ A substantial portion of these patients develops cirrhosis over long term and a subset of these patients develops hepatocellular carcinoma.^2^ Prevalence of hepatitis C in Pakistani population is 6.8% with up to 25% in rural areas.^3^ Prevalence of CKD based on health screening camps and community in Pakistan has been found to be around 12.5%-25%.^4^-^6^

There is a strong and causal relationship between chronic hepatitis C infection and kidney disease especially those of glomerular origin. Several glomerular diseases including mixed cryoglobulinemia, membranous nephropathy, membranoproliferative glomerulonephritis and polyarteritisnodosa are associated with hepatitis C.^7^

Several epidemiological studies have explored prevalence of hepatitis C in patients on hemodialysis. Prevalence of hepatitis C in hemodialysis patients in Pakistan is 23.7%-56.6%.^8^-^11^ However, there is limited local data on prevalence of hepatitis C in patients with CKD not yet on hemodialysis and international studies have shown variable results. Prevalence of hepatitis C antibody has ranged from 1.01% to 20% in patients with CKD in Peru, Spain, Turkey and Italy.^12^-^18^ Since prevalence of hepatitis C is high in general and hemodialysis population in Pakistan, its prevalence may be different in local CKD patients not yet on hemodialysis compared to international data.

The objective of this study was to determine the frequency of hepatitis C in hospitalized chronic kidney disease patients at a tertiary care center in Pakistan.

## METHODS

The study design was cross-sectional in nature. The study was approved by institutional review board. Patients between ages of 20-80 years with CKD not previously on renal replacement therapy (hemodialysis or peritoneal dialysis) and who were admitted to nephrology ward at a tertiary care facility were included. Informed consent was obtained from patients. CKD was defined as estimated GFR (eGFR) of less than <60ml/min/1.73m^2^ or persistent proteinuria by urinary dipstick for 3 or more months.^19^ eGFR was calculated by CKD-EPI formula as follows: GFR = 141 X min(Scr/κ,1)^α^ X max(Scr/κ,1)^-1.209^ X 0.993^Age^ X 1.018 [if female], Where Scr is serum creatinine (mg/dL), κ is 0.7 for females and 0.9 for males, α is –0.329 for females and –0.411 for males, min indicates the minimum of Scr/κ or 1, and max indicates the maximum of Scr/κ or 1.^20^

Sampling technique was non-probability consecutive sampling. Sample size of 171 was calculated with 95% confidence level, 6% margin of error and taking expected percentage of CKD patients with hepatitis C as 20%.

Patient’s history, medical records and laboratory information were reviewed to obtain data on patient’s age, sex, history of hypertension, diabetes mellitus, cardiovascular disease, serum creatinine, eGFR, and urine protein to creatinine ratio. Cardiovascular disease was defined as known prior history of coronary artery disease, cerebrovascular disease or peripheral vascular disease based on history and review of prior medical records. In addition, information was obtained regarding risk factors for hepatitis C including prior history of blood transfusion, IV drug use, prior surgery or whether patient is a known health care professional. Liver function tests were obtained in patients with hepatitis C positive status. Total bilirubin was measured by Jendrassik-Grof method. Alanine aminotransferase (ALT), aspartate aminotransferase (AST) and alkaline phosphatase (ALP) were measured using kinetic method. Hepatitis C was tested using 3^rd^ generation enzyme linked immunosorbent assay (ELISA). Hepatitis C RNA was tested by polymerase chain reaction (PCR) in patients with positive ELISA.

### Statistical Analysis

Continuous parametric variables were reported as means ± standard deviation; and categorical variables were expressed as percentages. Patients were divided into two groups based on presence of hepatitis C antibody by ELISA. Categorical variables were compared using the chi-square test, and continuous variables were compared using t-test. All statistical analyses were performed using SPSS 20.0 (Chicago, IL USA). For all tests, p values of <0.05 were considered statistically significant.

## RESULTS

A total of 180 patients were included in the study. Mean age of patients was 48.7±14.9 years. Of all patients, 105 (58.3%) were males and 75 (41.7%) were females, 139 (77.2%) were married, 32 (26.7%) were smokers, 31 (17.2%) had dyslipidemia, 152 (84.4%) had hypertension, 113 (62.8%) had diabetes mellitus and 26 (14.9%) had known cardiovascular disease. Mean eGFR of patients was 11.4±9.4 ml/min/1.73 m2. Of all patients, 134 (77.5%) had stage V CKD, 27 (15.6%) had stage IV CKD and 9 (5.2%) had stage III CKD.

Of all patients with CKD, 49 (27.2%) had hepatitis C test positive by ELISA. Hepatitis C PCR testing was done in 39 patients with hepatitis C ELISA positive status and 29 (74.4%) tested positive. Liver function tests were done in patients with positive hepatitis C antibody. Mean serum total bilirubin was 0.88±0.9 mg/dl, mean serum direct bilirubin was 0.50±0.70 mg/dl, mean ALT was 39.1±28.6 IU/L, mean AST was 34.7±12.3 IU/L, mean alkaline phosphatase was 240.8±77.7 IU/L. Proportion of patients with elevated ALT (i-e > 40 IU/L) was 29.4%. Characteristics of patients with and without hepatitis C antibody by ELISA are compared in Table-I.

**Table-I:** Comparison of characteristics of patients based on status of hepatitis C antibody by ELISA.

Characteristics	Patients with positive hepatitis C antibody by ELISA (N=49)	Patients with negative hepatitis C antibody by ELISA (N=131)	P value
Mean age (years)	48.7±13.8	48.7±15.4	0.99
Male Sex	61.2%	57.3%	0.63
Marital status	73.5%	78.6%	0.16
Hypertension	89.8%	83.7%	0.21
Diabetes Mellitus	75.0	60.2%	0.06
Cardiovascular Disease	18.4%	20.6%	0.76
Smokers	30.6%	22.8%	0.29
Mean duration of CKD (months)	9.9±3.9	7.4±2.1	0.72
H/O of IV drug use prior to hospitalization	10.2%	8.5%	0.72
H/O of blood transfusion	27.7%	26.0%	0.83
H/O of prior hospitalization	34.7%	29.2%	0.48
H/O of prior surgery	28.6%	25.3%	0.41
Health care worker	8.5%	4.7%	0.34

## DISCUSSION

Our study showed that frequency of hepatitis C antibody by ELISA in CKD patients was 27.2%. To our knowledge, this is the largest study of frequency of hepatitis C antibody in CKD patients not yet on hemodialysis in Pakistan.

In an old study in 1994 in Pakistan by Kumar H, frequency of hepatitis C antibody was 6% in 48 patients with CKD.^12^ However, this study is old and has very small sample size. International studies have shown variable results. In an Italian study of pre-dialysis CKD patients, incidence of hepatitis C was found to be 6.25% in 320 patients.^13^ In another study in Spain, hepatitis C antibody was present in 7.9% of 226 CKD patients with frequency of 13% in patients with creatinine clearance less than 30 ml/min.^14^ In a study by Fabrizi et al, hepatitis C antibody was present in 20% of CKD patients.^16^ In other studies, hepatitis C antibody was present in 3.9% and 7.0%.^17^,^18^ Variation in results is likely explained by variation in prevalence of hepatitis C in various geographic regions, different time periods of various studies, variation in methods for detecting hepatitis C antibody and practice of infection control measures in different countries. Though prevalence of hepatitis C is high in Pakistan, frequency of hepatitis C antibody in CKD patients in our study is significantly higher compared to general population. In various local studies in hospital setting in Pakistan, prevalence of hepatitis C has been found to be 3.3%-30.2% with most studies reporting higher prevalence compared to general population.^3^

Frequency of hepatitis C antibody in hemodialysis patients in Pakistan has been found to be around 23.7%-56.6%.^8^-^11^ In one study, hepatitis C antibody was present in 19.7% of patients even before start of hemodialysis.^10^ This implies, that high frequency of hepatitis C antibody in CKD patients prior to start of hemodialysis may in part contribute to high prevalence of hepatitis C in hemodialysis patients in Pakistan.

Use of hepatitis C antibody by ELISA may result in over-estimation of hepatitis C diagnosis, as some patients may have past infection with hepatitis C.^3^ In our study, hepatitis C PCR results were available for in 39 out of 49 patients due to loss of follow up. Hepatitis C PCR was positive in 74% of these patients, which is still significantly high compared to general population. We also couldn’t identify any specific risk factors for hepatitis C in our patient population, which may be due to relatively small sample size. Other studies in CKD population have identified low creatinine clearance^14^, history of blood transfusion,^17^,^18^ longer duration of CKD,^17^ history of intravenous drug use^18^ and elevated ALT levels^18^ to be associated with hepatitis C.

Our study results have several implications. First, strict universal infection control measures should be employed in hospital wards where CKD patients are admitted to prevent transmission of infection to other patients and health care workers. In addition, hepatitis C infection has been associated with greater risk of development and progression of CKD.^21^ Identification and treatment of hepatitis C in CKD patients may ameliorate progression of CKD, though this has to be verified through randomized controlled trials.

### Limitations of the study

Our study has several limitations. This is a single center study in hospitalized CKD patients only with limited sample size. Results may not be applicable to CKD patients seen in out-patient setting. In addition, we couldn’t identify any specific risk factor for hepatitis C infection in our CKD population. Hepatitis C PCR test results were available for majority but not all patients with hepatitis C ELISA antibody.

## CONCLUSION

Frequency of hepatitis C ELISA antibody is significantly high in hospitalized CKD patients. Strict universal infection control measures should be implemented in nephrology wards to prevent transmission of hepatitis C infection. Further studies are needed to see whether identification and treatment of hepatitis C in CKD patients will improve mortality or progression to end stage renal disease.
